# Transporting mitochondria in neurons

**DOI:** 10.12688/f1000research.7864.1

**Published:** 2016-07-18

**Authors:** Meredith M. Course, Xinnan Wang

**Affiliations:** 1 Department of Neurosurgery, Stanford University School of Medicine, Palo Alto, CA, USA; 2 Neurosciences Graduate Program, Stanford University, Stanford, CA, USA

**Keywords:** mitochondria, neurons, Transporting mitochondria, dynein, Myosins

## Abstract

Neurons demand vast and vacillating supplies of energy. As the key contributors of this energy, as well as primary pools of calcium and signaling molecules, mitochondria must be where the neuron needs them, when the neuron needs them. The unique architecture and length of neurons, however, make them a complex system for mitochondria to navigate. To add to this difficulty, mitochondria are synthesized mainly in the soma, but must be transported as far as the distant terminals of the neuron. Similarly, damaged mitochondria—which can cause oxidative stress to the neuron—must fuse with healthy mitochondria to repair the damage, return all the way back to the soma for disposal, or be eliminated at the terminals. Increasing evidence suggests that the improper distribution of mitochondria in neurons can lead to neurodegenerative and neuropsychiatric disorders. Here, we will discuss the machinery and regulatory systems used to properly distribute mitochondria in neurons, and how this knowledge has been leveraged to better understand neurological dysfunction.

## Introduction

The transport of mitochondria is critical to a neuron’s health. Although frequently referred to as “the powerhouse of the cell”, mitochondria do much more than produce ATP. In addition to being the cell’s major energy provider, mitochondria are responsible for storing and buffering Ca ^2+^, detoxifying ammonia, and producing some steroids ^1^, heme compounds ^2^, heat, and reactive oxygen species. They are vital to the metabolism of neurotransmitters glutamate and gamma-aminobutyric acid (GABA) ^3^, and send signals for apoptosis, proliferation, and cell survival ^4^. They even boast their own DNA and protein synthesis machinery as a vestige of their previous life as bacteria. It is thus unsurprising to learn that precise control of mitochondrial number, health, and distribution is especially critical to the neuron, which is a complex cell with high energy and regulatory demands.

Several features distinguish neurons from other cells. First, they have a long, thin axon—the longest axon in the human body can extend over one meter—and contain many areas of sub-specialization, like the pre-synapse, post-synapse, growth cones, and nodes of Ranvier ^5^, each with different metabolic needs. Second, as the carriers of synaptic information, neurons have ever-changing energy and Ca ^2+^ buffering demands, especially at their terminals. Finally, because neurons are post-mitotic and will stay with the organism for the duration of its life, they must be protected from excitotoxicity and kept in a state of homeostasis as long as possible. The appropriate allocation and sustenance of mitochondria are essential to fulfilling the many demands of the neuron, and keeping it in good health.

To meet the vacillating needs of neurons, about 30% to 40% of these spry organelles are in motion at any given time ^6– 9^. Properly distributing mitochondria throughout a neuron, however, is complicated by the fact that mitochondria are primarily produced in the soma, with most of their proteins encoded by nuclear DNA, but are needed as far away as the synaptic terminal. Static mitochondria pool at or near synapses, which may be important for rapid neuronal firing, while passing mitochondria may be recruited to support prolonged energy needs and repetitive neuronal firing ^10– 12^. Additionally, damaged mitochondria produce reactive oxygen species, which can be toxic to the cell, and these dysfunctional mitochondria must be repaired by fusing with new mitochondria transported from the soma, be returned to the soma for degradation in a process termed mitophagy, or be cleared through mitophagy in neurites *in situ* ^13^. Whether providing a service to the neuron, or needing clearance to prevent damage to the neuron, mitochondria must travel long distances and know precisely where and when to stop. When their transport machinery breaks down or signals regulating this machinery cannot be relayed, the consequence can be injury to or even death of the neuron ^9, 14– 17^. Here we will review the molecular mechanisms underlying mitochondrial transport in neurons, and what happens when they are disrupted.

## Transport machinery

Much like a train, organelle transport requires a track, a motor, and a cargo. For mitochondria—the cargo—the overwhelming majority of their tracks are microtubules, which in mammalian neurons have their plus ends oriented toward the axon terminal, and their minus ends toward the soma (although this homogeny is not the case in dendrites) ^18– 20^. This uniform polarity makes neuronal axons an especially useful model for studying organelle transport. The motors used to transport mitochondria depend on the direction in which they need to travel. In general, mitochondria move in the anterograde direction (away from the soma) using a family of kinesin motors, and move in the retrograde direction (toward the cell body) using the dynein motor ^21^. While kinesins and dynein are also used to carry other cargos, the motor adaptors that anchor the motor and cargo together are cargo-specific, allowing for the regulation of movement by particular cellular signals. In addition to microtubules, mitochondrial movement can be powered along actin filaments by myosin motors, a process that is required for short-range movement, and for opposing movement along the microtubules ^22– 24^.

### Anterograde movement with the kinesin heavy chain complex

**Figure 1.**
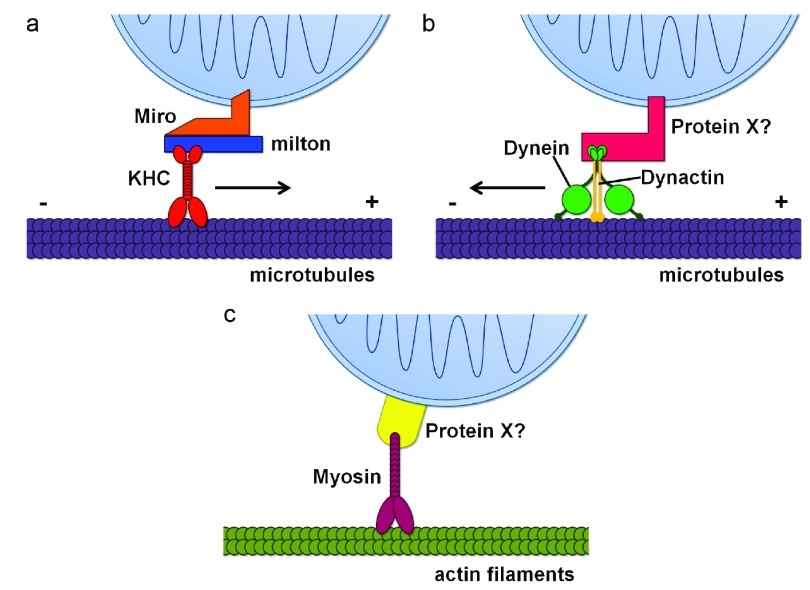
Schematic representation of mitochondrial transport machinery. ( **a**) The primary motor/adaptor complex mediating anterograde mitochondrial transport along microtubules (purple), including kinesin heavy chain (KHC) (red), Miro (orange), and milton (blue). ( **b**) The machinery mediating retrograde mitochondrial transport along microtubules (purple), including dynein (green), dynactin (gold), and a potential motor adaptor, Protein X (pink). Protein X could be the milton/Miro complex ^39^. ( **c**) Mitochondrial movement along actin filaments (olive), using a myosin motor (fuschia) and a potential motor adaptor, Protein X (yellow).

The best-characterized mitochondrial transport complex to date uses kinesin heavy chain (KHC, a member of the kinesin-1 family) as its motor, and Miro and milton as its motor adaptors. Miro stands for “mitochondrial Rho” and belongs to the atypical Rho (Ras homolog) family of GTPases (RhoT1/2 in mammals). Miro is anchored to the outer mitochondrial membrane (OMM) via its carboxy-terminus transmembrane domain. Miro binds to milton (trafficking protein, kinase-binding, or TRAK1/2 in mammals), which in turn binds to the carboxy-terminus of KHC ^25– 27^. Milton was identified in a *Drosophila* screen for blind flies and was named after the great poet and polemicist John Milton, who was also blind ^28^. Together, Miro and milton facilitate anterograde mitochondrial movement along microtubules by connecting mitochondria to KHC ( Figure 1a). When either Miro or milton is mutated in animal models, mitochondria are trapped in the soma and lose the ability to move out into the axons ^9, 14– 16, 26, 28^.

Miro and milton are not the only adaptors that can link mitochondria and KHC. Syntabulin can bind directly to the OMM and KHC, and disruption of syntabulin function has been shown to inhibit the anterograde transport of mitochondria in neurons ^29^. Similarly, disrupting fasciculation and elongation protein zeta-1 (FEZ1), and RAN-binding protein 2 (RANBP2) also affects mitochondrial distribution because of their association with kinesins, possibly KIF3A and KIF5B/C, respectively ^30– 34^.

Mutations in KHC have been shown to reduce anterograde mitochondrial movement but do not eliminate it entirely, which suggests that other kinesin motors may also play a role in anterograde mitochondrial motility ^21^. For example, kinesins from the Kinesin-3 family KIF1Bα and KLP6 may interact with KIF1 binding protein (KBP) to transport mitochondria ^35– 38^. KIF1B can transport mitochondria along microtubules *in vitro*, and mutations in *Klp6* inhibits anterograde mitochondrial motility into neurites; however, the roles of these other kinesins await further clarification.

### Retrograde movement with the dynein complex

Dynein is thought to act as the retrograde motor for microtubule-based mitochondrial movement, although far less is known about the mechanisms underlying its action. In contrast to the host of kinesins available for anterograde transport, there is only one identified dynein motor; however, dynein’s larger and more complex structure has made it difficult to study. Dynein has been shown to form a complex with dynactin, and this complex has been shown to also interact with milton/TRAK2 and with Miro ^39^, which lends support to dynein’s role in mitochondrial transport ( Figure 1b). Interestingly, dynein movement is also thought to depend on kinesin-1, as mutation in kinesin-1 reduces retrograde movement of mitochondria ^21^.

### Actin-based movement with myosin complexes

A small though not insignificant number of mitochondria are also transported along actin filaments ^22^. This is more common in actin-enriched neuronal compartments, like growth cones, dendritic spines, and synaptic boutons. Myosins are actin-based motors, and the myosin Myo19 has been shown to anchor directly to the OMM, and regulate mitochondrial motility ^24, 40^. Myosins V and VI have also been shown to play a role in mitochondrial motility by opposing microtubule-based mitochondrial transport ^23^, although whether these myosin motors attach directly to mitochondria or require a motor adaptor remains unknown ( Figure 1c). WAVE1 (WASP family verprolin homologous protein 1), which regulates actin polymerization, has been shown to be critical for mitochondrial transport in dendritic spines and filopodia—areas that are actin-rich—and therefore may be involved in the actin-based transport of mitochondria ^41^.

### Anchoring mitochondria

If 30% to 40% of mitochondria are in motion at any given time, then more than half of mitochondria are static. While understanding of how stationary pools of mitochondria are generated is still nascent, one protein, syntaphilin, stands out. Syntaphilin serves as a molecular brake, docking mitochondria by binding to both the mitochondrial surface and to the microtubule ^42^. Both kinesin-1 and the dynein light chain component LC8 have been shown to regulate this mechanism ^43, 44^. Intriguingly, a recent study using optogenetics has shown that the mitochondrial dance between mobility and stabilization depends on the balance of forces between motors and anchors, rather than all-or-none switching ^45^.

## Regulation of the kinesin heavy chain/milton/Miro complex

### Ca ^2+^

The ability of mitochondria to temporarily stop where they are needed is just as important as their ability to move. When cytosolic Ca ^2+^ concentration is elevated, Ca ^2+^ binds to the EF hands of Miro and triggers a transient and instantaneous conformational change in the KHC/milton/Miro complex. This conformational change causes dissociation of either the whole complex from microtubules ^9^, or KHC from mitochondria ^17^, which arrests movement of mitochondria. When Ca ^2+^ concentration is lowered, Ca ^2+^ is removed from Miro, and mitochondria are reattached to microtubules by the complex and can start moving again. The sensitivity of mitochondrial movement to Ca ^2+^ is likely a means by which mitochondria can be recruited to areas of high metabolic demand or low local ATP, like post-synaptic specializations and growth cones. During glutamate receptor activation, mitochondria are recruited where Ca ^2+^ influx is increased, which confers neurons with resistance to excitotoxicity ^9, 17^. Interestingly, brain-derived neurotrophic factor (BDNF) has recently been shown to arrest mitochondrial motility via Ca ^2+^ binding to Miro1 in cultured hippocampal neurons ^46^.

### Glucose

Glucose has recently been shown to influence mitochondrial motility via the KHC/milton/Miro complex. The small sugar UDP-GlcNAc is derived from glucose through the hexamine biosynthetic pathway. UDP-GlcNAc is affixed to milton by *O*-GlcNAc transferase (OGT), in a process called *O*-GlcNAcylation ^47^. Extracellular glucose concentration or OGT activity can modulate mitochondrial motility through *O*-GlcNAcylation of milton. This mechanism links nutrient availability to mitochondrial distribution, which could be a mechanism by which neurons maintain a balanced metabolic state.

### PINK1/Parkin

When mitochondria are severely damaged, they undergo mitophagy, a crucial cellular mechanism that eliminates depolarized mitochondria through autophagosomes and lysosomes. Damaged mitochondria must be stopped prior to the initiation of mitophagy. To accomplish this, mitochondrial depolarization activates PINK1 (PTEN-induced putative kinase 1)-mediated phosphorylation of Miro ^16, 48^, which subsequently triggers Parkin-dependent proteasomal degradation of Miro, thus releasing the mitochondria from its microtubule motors ^16, 49^. It is likely that stopping mitochondria in this manner is an early step in the quarantine of damaged mitochondria before degradation. In fact, this PINK1-mediated phosphorylation of Miro has been shown to protect dopaminergic neurons *in vivo* in *Drosophila* ^50^. PINK1 and Parkin can also work in concert to remove damaged mitochondria through local mitophagy in distal axons, which would obviate the need for the mitochondria to be transported all the way back to the soma, and instead require the recruitment of autophagosomes to the damaged mitochondria ^13^. How a cell chooses between transporting a mitochondrion back to the soma or using local mitophagy when it is damaged in the axon remains an outstanding question.

The dynamics of mitochondrial fission and fusion also plays a central role in PINK1/Parkin-mediated mitophagy. For example, mitofusin, a large GTPase that regulates mitochondrial fusion, is a target of the PINK1/Parkin pathway. Degradation of mitofusin prevents mitochondria from being able to fuse, and they instead fragment, a critical step prior to mitophagy ^51– 55^. An in-depth discussion of the role of mitochondrial dynamics in quality control merits its own review, and an excellent F1000 Faculty Review and two others are recommended in the References section ^56– 58^.

### Other milton/Miro regulators

In humans, milton is encoded by two different genes: *TRAK1* and *TRAK2*. It has been reported that TRAK1 binds to both kinesin-1 and dynein, while TRAK2 predominantly favors dynein ^39^. In *Drosophila*, milton has several splicing variants, one of which (milton-C) does not bind to KHC ^26^. These varying forms of milton may play an important role in regulating the KHC/milton/Miro complex.

Another regulator that merits mentioning is HUMMR (hypoxia up-regulated mitochondrial movement regulator), whose expression is induced by hypoxic conditions. HUMMR has been shown to interact with the KHC/milton/Miro complex, and increases the ratio of anterograde to retrograde movement of mitochondria ^59^. Similarly, a family of proteins encoded by an array of armadillo (Arm) repeat-containing genes has been shown to bind to milton/Miro and regulate mitochondrial motility ^60^.

It is worthwhile to note that mitochondrial fission and fusion also affect mitochondrial motility. The same mitofusin mentioned previously also binds to milton/Miro, and knockdown of mitofusin 2 has been shown to inhibit mitochondrial motility ^61^. Additionally, transient fusion has been shown to promote mitochondrial movement ^62^.

## Other regulators

The list of possible mitochondrial transport regulators burgeons daily, although thorough mechanisms remain scarce. For example, nerve growth factor can cause accumulation of mitochondria to its site of application ^63, 64^. Another growth factor, lysophosphatidic acid, can inhibit mitochondrial movement ^65^. Intracellular ATP levels regulate mitochondrial motility, which decreases when close to synapses, and local production of ADP can recruit more mitochondria to areas requiring more ATP ^66, 67^. Increased cAMP can increase mitochondrial motility ^68^. Pharmacological activation of AMP-activated protein kinase (AMPK) can promote anterograde movement of mitochondria for the formation of axon branches ^69^. Activation of the serotonin receptor increases mitochondrial movement via the AKT-GSK3β (Akt-glycogen synthase kinase 3β) pathway ^6^, and conversely, dopamine and activation of the dopamine receptor D2 can inhibit mitochondrial movement via the same AKT-GSK3β pathway ^70, 71^. One recent study shows that GSK-3β directly regulates dynein ^72^, while another study shows that it promotes anterograde movement ^68^. This list of molecules likely skims the surface of all the signals and sensors involved in mitochondrial motility, which are yet to be uncovered.

## Implications for neurological disorders

Because mitochondria are critical for energy production, calcium buffering, and cell survival pathways, it is not surprising to learn that impaired mitochondrial movement has been linked to neuronal dysfunction and neurological disorders ^73– 75^. The long distance travelled by mitochondria in neurons, as compared to in other cells, may account for the fact that neurons are more vulnerable to impairments in mitochondrial transport. Altered mitochondrial motility may provide an early indication of neuronal pathology prior to cell death, either because motility is directly affected or because it is altered as a consequence of other mitochondrial malfunctions.

### Neurodegenerative diseases

Aging itself has been shown to decrease neuronal mitochondrial motility in mice, and several age-dependent neurodegenerative diseases have been linked to mitochondrial motility defects ^76^. Mutations in the previously mentioned *PINK1* and *Parkin* are both causes of familial Parkinson’s disease (PD) ^77, 78^. In individuals lacking either functional PINK1 or Parkin, a failure to isolate, stop, and remove the damaged mitochondria may contribute to neuronal cell death. Unpublished work using patients’ samples from our laboratory also suggests that neurodegeneration in non- *PINK1/Parkin*-related PD cases may arise in a similar manner, and that stopping damaged mitochondrial motility is neuroprotective. This finding highlights the broader implications of mitochondrial motility in neuronal health and pathology.

The pathological forms of amyloid beta and tau, the chief markers of Alzheimer’s disease (AD), have both been shown to inhibit mitochondrial motility in several AD models ^79– 83^. Superoxide dismutase 1, soluble (SOD1), fused in sarcoma (FUS), C9orf72, and TAR DNA-binding protein 43 (TDP-43) mutations, which cause familial amyotrophic lateral sclerosis (also called Lou Gehrig’s disease), have also been shown to impair mitochondrial transport in mice, flies, and cultured neuronal models ^84– 91^. Mutant huntingtin protein, with the polyglutamine expansions characteristic of Huntington’s disease etiology, can act to “jam traffic” by mechanical obstruction, and may also bind to milton or even to the mitochondria itself to disrupt mitochondrial motility ^92– 94^. Mutations in *mitofusin 2* causing Charcot-Marie-Tooth disease alter mitochondria movement ^95^, and finally, mitochondrial motility defects have also been observed in models of hereditary spastic paraplegia, a disease characterized by axonal degeneration ^96, 97^.

### Neuropsychiatric disorders

A few psychiatric disorders have also been linked to mitochondrial motility defects. Mutations in disrupted in schizophrenia 1 ( *DISC1*) may contribute to both schizophrenia and some forms of depression ^98^. DISC1 complexes with TRAK1/milton and Miro1 to modulate anterograde transport of mitochondria ^99, 100^, and its interactors NDE1 and GSK3β have recently been recently shown to associate with TRAK1/milton and similarly play a role in mitochondrial motility ^68^. DISC1 also interacts with the previously mentioned FEZ1 ^101^, which binds to kinesins ^25, 28^. Among several causes, depression can be attributed to a loss of serotonin ^102^. Interestingly, the application of serotonin to cultured hippocampal neurons has been shown to increase mitochondrial motility ^6^.

## Closing remarks

The proper transport of mitochondria in neurons is critical to the homeostasis of the cell. Many questions in this field, however, remain to be answered. On a basic level of investigation, a more thorough understanding of the machinery—like the dynein motor, myosin motors, and the signals and adaptors that regulate this complex system—is still desperately needed.

A significant higher-level question is: how does the cell decide what to do with a damaged mitochondrion in the distal segment of an axon? The cell has several options: return the mitochondria to the soma for lysosomal degradation, which requires long-distance retrograde transport; degrade the mitochondrion via local mitophagy, which requires recruitment of autophagosomes to mitochondria and fusion of autophagosomes with lysosomes in the axon; or send a healthy mitochondrion from the soma via anterograde transport to repair the damage by fusing with the unhealthy mitochondrion. Could this decision be made on the basis of the nature or severity of the damage to the mitochondrion? Does this decision take into account the relative energy expended? What are the signals and molecules that execute this decision? These actions must also be influenced by the local metabolic state, *de novo* protein synthesis, and neuronal activity in extremities far from the cell body.

It is also crucial to explore the translational implications of these findings. What of this knowledge can be leveraged for therapeutic benefit? Perhaps mitochondrial motility could be used as a novel phenotypic readout to screen for more effective treatments for neurological disorders, as well as a way to diagnose the disease and monitor its progression. A more comprehensive understanding of the molecular mechanisms underlying mitochondrial transport will prove invaluable as it provides novel targets, like the KHC/milton/Miro complex, for diagnostic innovation and therapeutic intervention.

Most knowledge of mitochondrial movement in neurons has been uncovered using cultured rodent neurons. The application of emerging *in vivo* models will shed light on the physiological significance of the regulation of mitochondrial motility ^76, 103– 107^. Therefore, imaging mitochondria in living animals, especially during development and aging, as well as under disease conditions, will be an important step for the field.

Finally, given the inseparable relationship between neuronal function and metabolism, and mitochondrial motility and distribution, their underlying regulatory mechanisms must be interwoven. How do action potentials, neuronal signaling molecules like dopamine and serotonin, or metabolites like glucose, fatty acids, and amino acids influence mitochondrial motility and distribution? And how do mitochondrial motility and function reciprocally control neuronal homeostasis? Answers to these questions will reveal how neurons respond to changes in their activities and environments by regulating this cellular linchpin.

## References

[1] Rossier MF: T channels and steroid biosynthesis: in search of a link with mitochondria. *Cell Calcium.* 2006;40(2):155–64. 10.1016/j.ceca.2006.04.020 16759697

[2] Oh-hama T: Evolutionary consideration on 5-aminolevulinate synthase in nature. *Orig Life Evol Biosph.* 1997;27(4):405–12. 10.1023/A:1006583601341 9249985

[3] Kugler P Baier G: Mitochondrial enzymes related to glutamate and GABA metabolism in the hippocampus of young and aged rats: a quantitative histochemical study. *Neurochem Res.* 1992;17(2):179–85. 10.1007/BF00966797 1347164

[4] McBride HM Neuspiel M Wasiak S: Mitochondria: more than just a powerhouse. *Curr Biol.* 2006;16(14):R551–60. 10.1016/j.cub.2006.06.054 16860735

[5] Ohno N Kidd GJ Mahad D: Myelination and axonal electrical activity modulate the distribution and motility of mitochondria at CNS nodes of Ranvier. *J Neurosci.* 2011;31(20):7249–58. 10.1523/JNEUROSCI.0095-11.2011 21593309 PMC3139464

[6] Chen S Owens GC Crossin KL: Serotonin stimulates mitochondrial transport in hippocampal neurons. *Mol Cell Neurosci.* 2007;36(4):472–83. 10.1016/j.mcn.2007.08.004 17904380

[7] Overly CC Rieff HI Hollenbeck PJ: Organelle motility and metabolism in axons vs dendrites of cultured hippocampal neurons. *J Cell Sci.* 1996;109(Pt 5):971–80. 8743944 10.1242/jcs.109.5.971

[8] Waters J Smith SJ: Mitochondria and release at hippocampal synapses. *Pflugers Arch.* 2003;447(3):363–70. 10.1007/s00424-003-1182-0 14556074

[9] Wang X Schwarz TL: The mechanism of Ca ^2+^-dependent regulation of kinesin-mediated mitochondrial motility. *Cell.* 2009;136(1):163–74. 10.1016/j.cell.2008.11.046 19135897 PMC2768392

[10] Li Z Okamoto K Hayashi Y: The importance of dendritic mitochondria in the morphogenesis and plasticity of spines and synapses. *Cell.* 2004;119(6):873–87. 10.1016/j.cell.2004.11.003 15607982

[11] Verstreken P Ly CV Venken KJ: Synaptic mitochondria are critical for mobilization of reserve pool vesicles at *Drosophila* neuromuscular junctions. *Neuron.* 2005;47(3):365–78. 10.1016/j.neuron.2005.06.018 16055061

[12] Chang DT Honick AS Reynolds IJ: Mitochondrial trafficking to synapses in cultured primary cortical neurons. *J Neurosci.* 2006;26(26):7035–45. 10.1523/JNEUROSCI.1012-06.2006 16807333 PMC6673923

[13] Ashrafi G Schlehe JS LaVoie MJ: Mitophagy of damaged mitochondria occurs locally in distal neuronal axons and requires PINK1 and Parkin. *J Cell Biol.* 2014;206(5):655–70. 10.1083/jcb.201401070 25154397 PMC4151150

[14] Guo X Macleod GT Wellington A: The GTPase dMiro is required for axonal transport of mitochondria to *Drosophila* synapses. *Neuron.* 2005;47(3):379–93. 10.1016/j.neuron.2005.06.027 16055062

[15] Nguyen TT Oh SS Weaver D: Loss of Miro1-directed mitochondrial movement results in a novel murine model for neuron disease. *Proc Natl Acad Sci U S A.* 2014;111(35):E3631–40. 10.1073/pnas.1402449111 25136135 PMC4156725

[16] Wang X Winter D Ashrafi G: PINK1 and Parkin target Miro for phosphorylation and degradation to arrest mitochondrial motility. *Cell.* 2011;147(4):893–906. 10.1016/j.cell.2011.10.018 22078885 PMC3261796

[17] Macaskill AF Rinholm JE Twelvetrees AE: Miro1 is a calcium sensor for glutamate receptor-dependent localization of mitochondria at synapses. *Neuron.* 2009;61(4):541–55. 10.1016/j.neuron.2009.01.030 19249275 PMC2670979

[18] Baas PW Deitch JS Black MM: Polarity orientation of microtubules in hippocampal neurons: uniformity in the axon and nonuniformity in the dendrite. *Proc Natl Acad Sci U S A.* 1988;85(21):8335–9. 10.1073/pnas.85.21.8335 3054884 PMC282424

[19] Baas PW Black MM Banker GA: Changes in microtubule polarity orientation during the development of hippocampal neurons in culture. *J Cell Biol.* 1989;109(6 Pt 1):3085–94. 10.1083/jcb.109.6.3085 2592416 PMC2115969

[20] Heidemann SR Landers JM Hamborg MA: Polarity orientation of axonal microtubules. *J Cell Biol.* 1981;91(3 Pt 1):661–5. 6173385 10.1083/jcb.91.3.661 PMC2112798

[21] Pilling AD Horiuchi D Lively CM: Kinesin-1 and Dynein are the primary motors for fast transport of mitochondria in *Drosophila* motor axons. *Mol Biol Cell.* 2006;17(4):2057–68. 10.1091/mbc.E05-06-0526 16467387 PMC1415296

[22] Morris RL Hollenbeck PJ: Axonal transport of mitochondria along microtubules and F-actin in living vertebrate neurons. *J Cell Biol.* 1995;131(5):1315–26. 10.1083/jcb.131.5.1315 8522592 PMC2120647

[23] Pathak D Sepp KJ Hollenbeck PJ: Evidence that myosin activity opposes microtubule-based axonal transport of mitochondria. *J Neurosci.* 2010;30(26):8984–92. 10.1523/JNEUROSCI.1621-10.2010 20592219 PMC2904968

[24] Quintero OA DiVito MM Adikes RC: Human Myo19 is a novel myosin that associates with mitochondria. *Curr Biol.* 2009;19(23):2008–13. 10.1016/j.cub.2009.10.026 19932026 PMC2805763

[25] Fransson S Ruusala A Aspenström P: The atypical Rho GTPases Miro-1 and Miro-2 have essential roles in mitochondrial trafficking. *Biochem Biophys Res Commun.* 2006;344(2):500–10. 10.1016/j.bbrc.2006.03.163 16630562

[26] Glater EE Megeath LJ Stowers RS: Axonal transport of mitochondria requires milton to recruit kinesin heavy chain and is light chain independent. *J Cell Biol.* 2006;173(4):545–57. 10.1083/jcb.200601067 16717129 PMC2063864

[27] Giot L Bader JS Brouwer C: A protein interaction map of *Drosophila melanogaster*. *Science.* 2003;302(5651):1727–36. 10.1126/science.1090289 14605208

[28] Stowers RS Megeath LJ Górska-Andrzejak J: Axonal transport of mitochondria to synapses depends on milton, a novel *Drosophila* protein. *Neuron.* 2002;36(6):1063–77. 10.1016/S0896-6273(02)01094-2 12495622

[29] Cai Q Gerwin C Sheng ZH: Syntabulin-mediated anterograde transport of mitochondria along neuronal processes. *J Cell Biol.* 2005;170(6):959–69. 10.1083/jcb.200506042 16157705 PMC1804288

[30] Suzuki T Okada Y Semba S: Identification of FEZ1 as a protein that interacts with JC virus agnoprotein and microtubules: role of agnoprotein-induced dissociation of FEZ1 from microtubules in viral propagation. *J Biol Chem.* 2005;280(26):24948–56. 10.1074/jbc.M411499200 15843383

[31] Patil H Cho KI Lee J: Kinesin-1 and mitochondrial motility control by discrimination of structurally equivalent but distinct subdomains in Ran-GTP-binding domains of Ran-binding protein 2. *Open Biol.* 2013;3(3):120183. 10.1098/rsob.120183 23536549 PMC3718338

[32] Cho KI Cai Y Yi H: Association of the kinesin-binding domain of RanBP2 to KIF5B and KIF5C determines mitochondria localization and function. *Traffic.* 2007;8(12):1722–35. 10.1111/j.1600-0854.2007.00647.x 17887960

[33] Fujita T Maturana AD Ikuta J: Axonal guidance protein FEZ1 associates with tubulin and kinesin motor protein to transport mitochondria in neurites of NGF-stimulated PC12 cells. *Biochem Biophys Res Commun.* 2007;361(3):605–10. 10.1016/j.bbrc.2007.07.050 17669366

[34] Ikuta J Maturana A Fujita T: Fasciculation and elongation protein zeta-1 (FEZ1) participates in the polarization of hippocampal neuron by controlling the mitochondrial motility. *Biochem Biophys Res Commun.* 2007;353(1):127–32. 10.1016/j.bbrc.2006.11.142 17173861

[35] Nangaku M Sato-Yoshitake R Okada Y: KIF1B, a novel microtubule plus end-directed monomeric motor protein for transport of mitochondria. *Cell.* 1994;79(7):1209–20. 10.1016/0092-8674(94)90012-4 7528108

[36] Tanaka K Sugiura Y Ichishita R: KLP6: a newly identified kinesin that regulates the morphology and transport of mitochondria in neuronal cells. *J Cell Sci.* 2011;124(pt 4):2457–65. 10.1242/jcs.086470 21693574

[37] Wozniak MJ Melzer M Dorner C: The novel protein KBP regulates mitochondria localization by interaction with a kinesin-like protein. *BMC Cell Biol.* 2005;6:35. 10.1186/1471-2121-6-35 16225668 PMC1266353

[38] Lyons DA Naylor SG Mercurio S: KBP is essential for axonal structure, outgrowth and maintenance in zebrafish, providing insight into the cellular basis of Goldberg-Shprintzen syndrome. *Development.* 2008;135(3):599–608. 10.1242/dev.012377 18192286

[39] van Spronsen M Mikhaylova M Lipka J: TRAK/Milton motor-adaptor proteins steer mitochondrial trafficking to axons and dendrites. *Neuron.* 2013;77(3):485–502. 10.1016/j.neuron.2012.11.027 23395375

[40] Shneyer BI Ušaj M Henn A: Myo19 is an outer mitochondrial membrane motor and effector of starvation-induced filopodia. *J Cell Sci.* 2016;129(3):543–56. 10.1242/jcs.175349 26659663

[41] Sung JY Engmann O Teylan MA: WAVE1 controls neuronal activity-induced mitochondrial distribution in dendritic spines. *Proc Natl Acad Sci U S A.* 2008;105(8):3112–6. 10.1073/pnas.0712180105 18287015 PMC2268593

[42] Kang JS Tian JH Pan PY: Docking of axonal mitochondria by syntaphilin controls their mobility and affects short-term facilitation. *Cell.* 2008;132(1):137–48. 10.1016/j.cell.2007.11.024 18191227 PMC2259239

[43] Chen YM Gerwin C Sheng ZH: Dynein light chain LC8 regulates syntaphilin-mediated mitochondrial docking in axons. *J Neurosci.* 2009;29(30):9429–38. 10.1523/JNEUROSCI.1472-09.2009 19641106 PMC6666546

[44] Chen Y Sheng ZH: Kinesin-1-syntaphilin coupling mediates activity-dependent regulation of axonal mitochondrial transport. *J Cell Biol.* 2013;202(2):351–64. 10.1083/jcb.201302040 23857772 PMC3718985

[45] van Bergeijk P Adrian M Hoogenraad CC: Optogenetic control of organelle transport and positioning. *Nature.* 2015;518(7537):111–4. 10.1038/nature14128 25561173 PMC5063096

[46] Su B Ji YS Sun XL: Brain-derived neurotrophic factor (BDNF)-induced mitochondrial motility arrest and presynaptic docking contribute to BDNF-enhanced synaptic transmission. *J Biol Chem.* 2014;289(3):1213–26. 10.1074/jbc.M113.526129 24302729 PMC3894308

[47] Pekkurnaz G Trinidad JC Wang X: Glucose regulates mitochondrial motility via Milton modification by *O*-GlcNAc transferase. *Cell.* 2014;158(1):54–68. 10.1016/j.cell.2014.06.007 24995978 PMC4224014

[48] Lai YC Kondapalli C Lehneck R: Phosphoproteomic screening identifies Rab GTPases as novel downstream targets of PINK1. *EMBO J.* 2015;34(22):2840–61. 10.15252/embj.201591593 26471730 PMC4654935

[49] Liu S Sawada T Lee S: Parkinson's disease-associated kinase PINK1 regulates Miro protein level and axonal transport of mitochondria. *PLoS Genet.* 2012;8(3):e1002537. 10.1371/journal.pgen.1002537 22396657 PMC3291531

[50] Tsai PI Course MM Lovas JR: PINK1-mediated phosphorylation of Miro inhibits synaptic growth and protects dopaminergic neurons in *Drosophila*. *Sci Rep.* 2014;4: 6962. 10.1038/srep06962 25376463 PMC4223694

[51] Chan NC Salazar AM Pham AH: Broad activation of the ubiquitin-proteasome system by Parkin is critical for mitophagy. *Hum Mol Genet.* 2011;20(9):1726–37. 10.1093/hmg/ddr048 21296869 PMC3071670

[52] Chen Y Dorn GW 2nd: PINK1-phosphorylated mitofusin 2 is a Parkin receptor for culling damaged mitochondria. *Science.* 2013;340(6131):471–5. 10.1126/science.1231031 23620051 PMC3774525

[53] Poole AC Thomas RE Yu S: The mitochondrial fusion-promoting factor mitofusin is a substrate of the PINK1/parkin pathway. *PLoS One.* 2010;5(4):e10054. 10.1371/journal.pone.0010054 20383334 PMC2850930

[54] Tanaka A Cleland MM Xu S: Proteasome and p97 mediate mitophagy and degradation of mitofusins induced by Parkin. *J Cell Biol.* 2010;191(7):1367–80. 10.1083/jcb.201007013 21173115 PMC3010068

[55] Ziviani E Tao RN Whitworth AJ: *Drosophila* parkin requires PINK1 for mitochondrial translocation and ubiquitinates mitofusin. *Proc Natl Acad Sci U S A.* 2010;107(11):5018–23. 10.1073/pnas.0913485107 20194754 PMC2841909

[56] Pickrell AM Youle RJ: The roles of PINK1, parkin, and mitochondrial fidelity in Parkinson's disease. *Neuron.* 2015;85(2):257–73. 10.1016/j.neuron.2014.12.007 25611507 PMC4764997

[57] Narendra D Walker JE Youle R: Mitochondrial quality control mediated by PINK1 and Parkin: links to parkinsonism. *Cold Spring Harb Perspect Biol.* 2012;4(11): pii: a011338. 10.1101/cshperspect.a011338 23125018 PMC3536340

[58] Kornmann B: Quality control in mitochondria: use it, break it, fix it, trash it. *F1000Prime Rep.* 2014;6:15. 10.12703/P6-15 24669296 PMC3944741

[59] Li Y Lim S Hoffman D: HUMMR, a hypoxia- and HIF-1alpha-inducible protein, alters mitochondrial distribution and transport. *J Cell Biol.* 2009;185(6):1065–81. 10.1083/jcb.200811033 19528298 PMC2711615

[60] López-Doménech G Serrat R Mirra S: The Eutherian *Armcx* genes regulate mitochondrial trafficking in neurons and interact with Miro and Trak2. *Nat Commun.* 2012;3:814. 10.1038/ncomms1829 22569362

[61] Misko A Jiang S Wegorzewska I: Mitofusin 2 is necessary for transport of axonal mitochondria and interacts with the Miro/Milton complex. *J Neurosci.* 2010;30(12):4232–40. 10.1523/JNEUROSCI.6248-09.2010 20335458 PMC2852190

[62] Liu X Weaver D Shirihai O: Mitochondrial 'kiss-and-run': interplay between mitochondrial motility and fusion-fission dynamics. *EMBO J.* 2009;28(20):3074–89. 10.1038/emboj.2009.255 19745815 PMC2771091

[63] Chada SR Hollenbeck PJ: Mitochondrial movement and positioning in axons: the role of growth factor signaling. *J Exp Biol.* 2003;206(Pt 12):1985–92. 10.1242/jeb.00263 12756280

[64] Chada SR Hollenbeck PJ: Nerve growth factor signaling regulates motility and docking of axonal mitochondria. *Curr Biol.* 2004;14(14):1272–6. 10.1016/j.cub.2004.07.027 15268858

[65] Minin AA Kulik AV Gyoeva FK: Regulation of mitochondria distribution by RhoA and formins. *J Cell Sci.* 2006;119(Pt 4):659–70. 10.1242/jcs.02762 16434478

[66] Mironov SL: ADP regulates movements of mitochondria in neurons. *Biophys J.* 2007;92(8):2944–52. 10.1529/biophysj.106.092981 17277190 PMC1831680

[67] Mironov SL: Complexity of mitochondrial dynamics in neurons and its control by ADP produced during synaptic activity. *Int J Biochem Cell Biol.* 2009;41(10):2005–14. 10.1016/j.biocel.2009.04.009 19379829

[68] Ogawa F Murphy LC Malavasi EL: NDE1 and GSK3β Associate with TRAK1 and Regulate Axonal Mitochondrial Motility: Identification of Cyclic AMP as a Novel Modulator of Axonal Mitochondrial Trafficking. *ACS Chem Neurosci.* 2016;7(5):553–64. 10.1021/acschemneuro.5b00255 26815013

[69] Tao K Matsuki N Koyama R: AMP-activated protein kinase mediates activity-dependent axon branching by recruiting mitochondria to axon. *Dev Neurobiol.* 2014;74(6):557–73. 10.1002/dneu.22149 24218086

[70] Morfini G Szebenyi G Elluru R: Glycogen synthase kinase 3 phosphorylates kinesin light chains and negatively regulates kinesin-based motility. *EMBO J.* 2002;21(3):281–93. 10.1093/emboj/21.3.281 11823421 PMC125832

[71] Chen S Owens GC Edelman DB: Dopamine inhibits mitochondrial motility in hippocampal neurons. *PLoS One.* 2008;3(7):e2804. 10.1371/journal.pone.0002804 18665222 PMC2467486

[72] Gao FJ Hebbar S Gao XA: GSK-3β Phosphorylation of Cytoplasmic Dynein Reduces Ndel1 Binding to Intermediate Chains and Alters Dynein Motility. *Traffic.* 2015;16(9):941–61. 10.1111/tra.12304 26010407 PMC4543430

[73] de Vos KJ Grierson AJ Ackerley S: Role of axonal transport in neurodegenerative diseases. *Annu Rev Neurosci.* 2008;31:151–73. 10.1146/annurev.neuro.31.061307.090711 18558852

[74] Morfini GA Burns M Binder LI: Axonal transport defects in neurodegenerative diseases. *J Neurosci.* 2009;29(41):12776–86. 10.1523/JNEUROSCI.3463-09.2009 19828789 PMC2801051

[75] Deheshi S Pasqualotto BA Rintoul GL: Mitochondrial trafficking in neuropsychiatric diseases. *Neurobiol Dis.* 2013;51:66–71. 10.1016/j.nbd.2012.06.015 22750523

[76] Gilley J Seereeram A Ando K: Age-dependent axonal transport and locomotor changes and tau hypophosphorylation in a "P301L" tau knockin mouse. *Neurobiol Aging.* 2012;33(3):621.e1–621.e15. 10.1016/j.neurobiolaging.2011.02.014 21492964

[77] Valente EM Abou-Sleiman PM Caputo V: Hereditary early-onset Parkinson's disease caused by mutations in *PINK1*. *Science.* 2004;304(5674):1158–60. 10.1126/science.1096284 15087508

[78] Kitada T Asakawa S Hattori N: Mutations in the *parkin* gene cause autosomal recessive juvenile parkinsonism. *Nature.* 1998;392(6676):605–8. 10.1038/33416 9560156

[79] Pigino G Morfini G Pelsman A: Alzheimer's presenilin 1 mutations impair kinesin-based axonal transport. *J Neurosci.* 2003;23(11):4499–508. 12805290 10.1523/JNEUROSCI.23-11-04499.2003 PMC6740780

[80] Stokin GB Lillo C Falzone TL: Axonopathy and transport deficits early in the pathogenesis of Alzheimer's disease. *Science.* 2005;307(5713):1282–8. 10.1126/science.1105681 15731448

[81] Rui Y Tiwari P Xie Z: Acute impairment of mitochondrial trafficking by beta-amyloid peptides in hippocampal neurons. *J Neurosci.* 2006;26(41):10480–7. 10.1523/JNEUROSCI.3231-06.2006 17035532 PMC6674697

[82] Vossel KA Zhang K Brodbeck J: Tau reduction prevents Abeta-induced defects in axonal transport. *Science.* 2010;330(6001):198. 10.1126/science.1194653 20829454 PMC3024010

[83] Calkins MJ Reddy PH: Amyloid beta impairs mitochondrial anterograde transport and degenerates synapses in Alzheimer's disease neurons. *Biochim Biophys Acta.* 2011;1812(4):507–13. 10.1016/j.bbadis.2011.01.007 21241801 PMC3042500

[84] Sasaki S Iwata M: Impairment of fast axonal transport in the proximal axons of anterior horn neurons in amyotrophic lateral sclerosis. *Neurology.* 1996;47(2):535–40. 10.1212/WNL.47.2.535 8757033

[85] De Vos KJ Chapman AL Tennant ME: Familial amyotrophic lateral sclerosis-linked SOD1 mutants perturb fast axonal transport to reduce axonal mitochondria content. *Hum Mol Genet.* 2007;16(22):2720–8. 10.1093/hmg/ddm226 17725983 PMC4516806

[86] Magrané J Hervias I Henning MS: Mutant SOD1 in neuronal mitochondria causes toxicity and mitochondrial dynamics abnormalities. *Hum Mol Genet.* 2009;18(23):4552–64. 10.1093/hmg/ddp421 19779023 PMC2773270

[87] Baldwin KR Godena VK Hewitt VL: Axonal transport defects are a common phenotype in *Drosophila* models of ALS. *Hum Mol Genet.* 2016;pii: ddw105. 10.1093/hmg/ddw105 27056981 PMC5181624

[88] Magrané J Cortez C Gan WB: Abnormal mitochondrial transport and morphology are common pathological denominators in SOD1 and TDP43 ALS mouse models. *Hum Mol Genet.* 2014;23(6):1413–24. 10.1093/hmg/ddt528 24154542 PMC3929084

[89] Shi P Ström AL Gal J: Effects of ALS-related SOD1 mutants on dynein- and KIF5-mediated retrograde and anterograde axonal transport. *Biochim Biophys Acta.* 2010;1802(9):707–16. 10.1016/j.bbadis.2010.05.008 20510358 PMC2907440

[90] Shan X Chiang PM Price DL: Altered distributions of Gemini of coiled bodies and mitochondria in motor neurons of *TDP-43* transgenic mice. *Proc Natl Acad Sci U S A.* 2010;107(37):16325–30. 10.1073/pnas.1003459107 20736350 PMC2941282

[91] Bosco DA Morfini G Karabacak NM: Wild-type and mutant SOD1 share an aberrant conformation and a common pathogenic pathway in ALS. *Nat Neurosci.* 2010;13(11):1396–403. 10.1038/nn.2660 20953194 PMC2967729

[92] Trushina E Dyer RB Badger JD 2nd: Mutant huntingtin impairs axonal trafficking in mammalian neurons *in vivo* and *in vitro*. *Mol Cell Biol.* 2004;24(18):8195–209. 10.1128/MCB.24.18.8195-8209.2004 15340079 PMC515048

[93] Chang DT Rintoul GL Pandipati S: Mutant huntingtin aggregates impair mitochondrial movement and trafficking in cortical neurons. *Neurobiol Dis.* 2006;22(2):388–400. 10.1016/j.nbd.2005.12.007 16473015

[94] Orr AL Li S Wang CE: N-terminal mutant huntingtin associates with mitochondria and impairs mitochondrial trafficking. *J Neurosci.* 2008;28(11):2783–92. 10.1523/JNEUROSCI.0106-08.2008 18337408 PMC2652473

[95] Baloh RH Schmidt RE Pestronk A: Altered axonal mitochondrial transport in the pathogenesis of Charcot-Marie-Tooth disease from mitofusin 2 mutations. *J Neurosci.* 2007;27(2):422–30. 10.1523/JNEUROSCI.4798-06.2007 17215403 PMC6672077

[96] Ferreirinha F Quattrini A Pirozzi M: Axonal degeneration in paraplegin-deficient mice is associated with abnormal mitochondria and impairment of axonal transport. *J Clin Invest.* 2004;113(2):231–42. 10.1172/JCI20138 14722615 PMC311437

[97] Kasher PR De Vos KJ Wharton SB: Direct evidence for axonal transport defects in a novel mouse model of mutant spastin-induced hereditary spastic paraplegia (HSP) and human HSP patients. *J Neurochem.* 2009;110(1):34–44. 10.1111/j.1471-4159.2009.06104.x 19453301

[98] Millar JK Wilson-Annan JC Anderson S: Disruption of two novel genes by a translocation co-segregating with schizophrenia. *Hum Mol Genet.* 2000;9(9):1415–23. 10.1093/hmg/9.9.1415 10814723

[99] Atkin TA MacAskill AF Brandon NJ: Disrupted in Schizophrenia-1 regulates intracellular trafficking of mitochondria in neurons. *Mol Psychiatry.* 2011;16(2):122–4, 121. 10.1038/mp.2010.110 21079610

[100] Ogawa F Malavasi EL Crummie DK: DISC1 complexes with TRAK1 and Miro1 to modulate anterograde axonal mitochondrial trafficking. *Hum Mol Genet.* 2014;23(4):906–19. 10.1093/hmg/ddt485 24092329 PMC3900104

[101] Miyoshi K Honda A Baba K: *Disrupted-In-Schizophrenia 1*, a candidate gene for schizophrenia, participates in neurite outgrowth. *Mol Psychiatry.* 2003;8(7):685–94. 10.1038/sj.mp.4001352 12874605

[102] Belmaker RH Agam G: Major depressive disorder. *N Engl J Med.* 2008;358(1):55–68. 10.1056/NEJMra073096 18172175

[103] Takihara Y Inatani M Eto K: *In vivo* imaging of axonal transport of mitochondria in the diseased and aged mammalian CNS. *Proc Natl Acad Sci U S A.* 2015;112(33):10515–20. 10.1073/pnas.1509879112 26240337 PMC4547257

[104] Misgeld T Kerschensteiner M Bareyre FM: Imaging axonal transport of mitochondria *in vivo*. *Nat Methods.* 2007;4(7):559–61. 10.1038/nmeth1055 17558414

[105] Bolea I Gan WB Manfedi G: Imaging of mitochondrial dynamics in motor and sensory axons of living mice. *Methods Enzymol.* 2014;547:97–110. 10.1016/B978-0-12-801415-8.00006-0 25416354

[106] Wang X Schwarz TL: Imaging axonal transport of mitochondria. *Methods Enzymol.* 2009;457:319–33. 10.1016/S0076-6879(09)05018-6 19426876 PMC2996865

[107] Plucinska G Paquet D Hruscha A: *In vivo* imaging of disease-related mitochondrial dynamics in a vertebrate model system. *J Neurosci.* 2012;32(46):16203–12. 10.1523/JNEUROSCI.1327-12.2012 23152604 PMC6794024

